# Vaccine Criticism on the World Wide Web

**DOI:** 10.2196/jmir.7.2.e17

**Published:** 2005-06-29

**Authors:** Richard K Zimmerman, Robert M Wolfe, Dwight E Fox, Jake R Fox, Mary Patricia Nowalk, Judith A Troy, Lisa K Sharp

**Affiliations:** ^3^Department of Family MedicineFeinberg School of MedicineNorthwestern UniversityChicagoILUSA; ^2^Department of Behavioral and Community Health SciencesUniversity of Pittsburgh Graduate School of Public HealthPittsburghPAUSA; ^1^Department of Family Medicine and Clinical EpidemiologyUniversity of Pittsburgh School of MedicinePittsburghPAUSA

**Keywords:** Vaccines, Internet, immunization, vaccine safety, vaccine criticism, anti-vaccine

## Abstract

**Background:**

The incidence of vaccine-preventable diseases is directly related to the number of unvaccinated children. Parents who refuse vaccination of their children frequently express concerns about vaccine safety. The Internet can influence perceptions about vaccines because it is the fastest growing source of consumer health information. However, few studies have analyzed vaccine criticism on the Web.

**Objective:**

The purposes of this paper are to examine vaccine criticism on the Internet and to analyze the websites in order to identify common characteristics and ethical allegations.

**Methods:**

A structured Web search was conducted for the terms “vaccine,” “vaccination,” “vaccinate,” and “anti-vaccination” using a metasearch program that incorporated 8 search engines. This yielded 1138 Web pages representing 750 sites. Two researchers reviewed the sites for inclusion/exclusion criteria, resulting in 78 vaccine-critical sites, which were then abstracted for design, content, and allegations.

**Results:**

The most common characteristic of vaccine-critical websites was the inclusion of statements linking vaccinations with specific adverse reactions, especially idiopathic chronic diseases such as multiple sclerosis, autism, and diabetes. Other common attributes (≥ 70% of websites) were links to other vaccine-critical websites; charges that vaccines contain contaminants, mercury, or “hot lots” that cause adverse events; claims that vaccines provide only temporary protection and that the diseases prevented are mild; appeals for responsible parenting through education and resisting the establishment; allegations of conspiracies and cover-ups to hide the truth about vaccine safety; and charges that civil liberties are violated through mandatory vaccination.

**Conclusions:**

Vaccine-critical websites frequently make serious allegations. With the burgeoning of the Internet as a health information source, an undiscerning or incompletely educated public may accept these claims and refuse vaccination of their children. As this occurs, the incidence of vaccine-preventable diseases can be expected to rise.

## Introduction

The number of unvaccinated children is rising in the United States; the estimated number of unvaccinated children aged 19 to 35 months increased from 14719 in 1995 to 24073 in 2000 [1]. The number of unvaccinated children plays an important role in the incidence of vaccine-preventable diseases. For example, the frequency of abstainers from vaccination has been associated with the incidence of measles and pertussis among both vaccinated and unvaccinated children [2].

### Parental Concerns About Vaccine Safety

A number of studies have documented parental concerns about vaccine safety [1,3-5]. A 2004 online survey showed that half of parents are concerned that a child might develop a long-term medical condition as a result of vaccination [6]. One tenth of parents are uncomfortable having their child vaccinated due to health concerns [6]. Another US national survey found that the majority of parents of young children support the use of immunization, but about one quarter are concerned that children receive more vaccines than are good for them, and that, as a result, their immune systems could be weakened [7]. About one fifth (19%) do not think vaccines are proven safe prior to use in the United States [7]. A third study comparing responses from parents of unvaccinated versus vaccinated children found that parents of the unvaccinated were significantly more likely to ask that their child not be vaccinated, to believe that the MMR (measles, mumps, rubella) vaccine causes autism, to be concerned about side effects, and to believe that children receive too many vaccines [4]. In Colorado, the percentage of children with philosophical exemptions to immunization increased from 1.02% to 1.87% from 1987 to 1998 [2]. Thus, many parents are concerned about vaccine safety, and a growing number are expressing this by refusing vaccination of their children.

The media, both print and electronic, are frequently used to educate the public about health issues. Similarly, the media have been used to discourage uptake of known public health measures such as vaccination. For example, an international study examined anti-vaccine campaigns in the media, pertussis vaccine coverage, and disease incidence in the United States and several European countries [8]. Those countries with concerted anti-vaccine campaigns as reported in contemporary news stories had significantly higher incidence of pertussis compared with countries with few or no media reports on alleged vaccine adverse events. The latter countries, in general, maintained high vaccination levels with low disease incidence.

### Influence of the Internet and Purpose to Study Vaccine-Critical Websites

The Internet, the newest electronic news medium, has the potential to influence perceptions about vaccines because it is the fastest growing source of consumer health information. In fact, most (67%) US adults use the Internet, and of these, 40% to 80% use it to access health information [9-11]. With the rapid expansion of the Internet (an estimated 19000 websites in 1995 to 36 million websites in 2001[12]) and the increasing number of people seeking health information on the Web (an estimated 110 million adults[10]), frequent updates of the health information being disseminated via the Internet are necessary.

The vaccine criticism movement has taken advantage of the Internet and its ability to reach parents seeking information on vaccines and vaccine safety. Parents can find this information with just a few key strokes. Three studies, conducted from 1999 to 2001, provide some insight into the vaccine criticism movement on the Internet, describing the content and design attributes of “anti-vaccination” websites [13-15]. The purpose of this paper is to more broadly examine vaccine criticism on the Internet in 2004 and update previous findings. This is the largest study of such websites conducted in the United States to date. This update will enable health providers to better understand the arguments against vaccination and the questions regarding vaccination that parents and patients may present to them.

## Methods

Web pages about vaccination were identified using Copernic Agent Professional version 6.11 (Copernic Technologies Inc, Saine-Foy, Quebec, Canada), which is an Internet search program designed to simultaneously submit searches on numerous engines and return unduplicated results. The search engines used were AltaVista, FAST Search (alltheweb.com), Google (which also powers Yahoo! and AOL), HotBot, Lycos, MSN Web search, Netscape Netcenter, and Teoma. The search was conducted on December 5, 2003, using the terms “vaccine,” “vaccination,” “vaccinate,” and “anti-vaccination.” Previous research showed that sites critical of vaccination were much more likely to be found with these terms rather than “immunization” [15]. The result was 1138 Web pages representing 750 sites.

The exclusion criteria were as follows: (1) listserv or newsgroup containing online conversation; (2) information applicable primarily to animals; (3) posts of brief notices about content on other sites; (4) online commercial news service, health/medical journal, or library; (5) non-English language site; 6) exclusively adult immunization; and (7) inactive links. The inclusion criterion was content encouraging vaccine refusal or emphasizing the dangers of vaccines.

Two researchers independently reviewed the sites and agreed that 662 were excluded and 22 were included, but they disagreed on 66 sites. A third reviewer reviewed these and determined inclusion or exclusion, leading to a final count of 78 sites.

### Data Collection/Website Review

The websites meeting the exclusion and inclusion criteria were downloaded in 2004 onto a CD using Aeria Leech 3.3 software (Tampa, FL), which downloads Web content. In this way, all the reviewers accessed identical information, as content of the websites may change over time. Criteria for evaluation of the sites were adapted from published criteria for evaluating health related websites, design and attribute characteristics used in previous studies in 2000 (eg, links to other vaccine-critical websites and sale of books, tapes, CDs from the site), specific vaccine safety concerns (eg, association with autism, multiple sclerosis), and ethical allegations (eg, conspiracy, civil liberty violations) [13,16,17]. A list of variables was defined and, after data collection, was refined. In particular, fifty variables were defined in detail to minimize interpretation differences. For each variable, 2 reviewers (1 clinician and 1 social scientist) independently examined all pages of each website to determine if the attributes were present (coded as 1) or absent (coded as 0).

### Data Analysis

Interrater reliability for each variable was determined using the kappa statistic. Variables with a kappa value less than 0.5, indicating a low level of agreement between the two reviewers, were not included in further analyses (4 variables). Of the remaining 46 variables, 16 were retained as collected, and 30 were combined into 12 variables using logical groupings (eg, sites promoting alternative therapies, herbal remedies, or homeopathy as adequate protection against infectious disease were combined). For combined variables, if a website was found to have at least one of the individual attributes present, then the combined variable was coded as being present for that website. The kappa statistic was calculated for the combined variables.

To determine the percentage of websites containing each of the attributes, it was necessary to average the two reviewers' coded values (ie, if both reviewers coded an attribute as present, the average was 1; if one reviewer coded the attribute as present and one reviewer coded it as absent, the average was 0.5; and if both coded the attribute as absent, the average was 0). These scores were summed and divided by the total number of websites.

Variable groupings were then created by combining the 28 variables into the following clusters: promotion of vaccine criticism, emotive appeals, alternative medicine, disease risk/vaccine safety, and ethical allegations. Spearman correlations compared total percent presence of attributes in each cluster to assess whether certain groups of attributes were frequently found together in vaccine-critical websites. Analyses were performed using SPSS 12.0 (SPSS Inc, Chicago, IL).

## Results

In total, 78 websites were reviewed. Table 2 lists the website characteristics, the frequency with which they appeared, and the interrater reliability for website reviews. The single most common characteristic of vaccine-critical websites was the inclusion of statements linking vaccinations with specific adverse reactions, especially idiopathic chronic diseases such as multiple sclerosis, autism, and diabetes.

Other common (≥ 75% of websites) characteristics were links to other vaccine-critical websites, charges that vaccines contain contaminants that cause adverse events, allegations of conspiracies to hide the truth about vaccine safety and efficacy, appeals for responsible parenting through education and resisting the establishment, and claims that vaccines provide only temporary protection and are therefore not worth the risk. Examples of the types of vaccine criticism on the Internet are provided in Table 1.

**Table 1:** Types and examples of vaccine criticism on the Web

**Type of Information**	**Example**
Promotion of vaccine criticism	“Then one word can describe this new video, 'Vaccines: What CDC Documents and Science Reveal,' by world-renowned vaccine expert Dr. Sherri Tenpenny: essential. To put it simply, if you are dedicated to protecting and enhancing your life, your family's, or your patients', but you have not been exposed to the often startling but thoroughly documented information in this video, there is a dangerous gap in your knowledge. Whether you have explored the issue of the dangers of vaccines extensively or not at all, I more than recommend you watch this video—I implore you to do so. Available on VHS. Just $24.95.” (www.mercola.com/forms/vaccine_video.htm)
Alternative medicine	“Homeopathic Medicine for counteracting the effects of vaccination: while not as good as NOT getting vaccinated, I have been told by a number of healers that the homeopathic medicine Thuja was very helpful.” (www.relfe.com/vaccine.html) “For those that decide not to immunize their children, naturopathic medicine does offer several alternatives. For those that wish to have some sort of protection, there are homeopathic mixtures of the vaccines which can be used. Constitutional homeopathy can also be used to strengthen the vital force of your children.” (www.naturdoctor.com/Chapters/Articles/vaccinate.html)
Emotive appeals	“I helplessly watched my daughter suffer an excruciatingly slow death as she screamed and arched her back in pain, while the vaccine did as it was intended to do and assaulted her immature immune system. The poisons used as preservatives seeped through her tiny body, overwhelming her vital organs one by one until they collapsed. It is an image that will haunt me forever and I hope no other parent ever has to witness it. A death sentence considered too inhumane for this county's most violent criminals was handed down to my beautiful, innocent, infant daughter, death by lethal injection.” (www.mercola.com/2002/aug/7/vaccine_death.htm)
Vaccine safety	“Vaccination causes significant death and disability at an astounding personal and financial cost to families and taxpayers.” (www.relfe.com/vaccine.html) “Personal stories of vaccine damage, as told by sad parents who lost a child to the shots, remind us that real families, and real children, are being affected.” (http://thinktwice.com/risk.htm)
Ethical allegations	“Adverse reactions to vaccines are more common than many people realize. In fact, the US government operates a secret database that contains the names of several thousand children who were healthy and alive just prior to receiving the vaccines.” (http://thinktwice.com/risk.htm)

**Table 2:** Types of information on vaccine-critical websites

**Type of Information**	**Websites With** **This Attribute (%)**	**Interrater Reliability ** (Kappa)*
**DESCRIPTIVE CONTENT**		
**Vaccine Criticism**		
Links to other sites critical of vaccines	80	0.718
Information for legally avoiding immunizations	47	0.719
Information on reporting adverse events	35	0.661
Vaccine critical books, tapes, compact discs for purchase from site	33	0.738
Email listserv or chat room (eg, to discuss vaccine dangers)	28	0.558
Solicitations for contributions for website support or anti-vaccine cause or organization	21	0.654
Links to attorneys	20	0.800
**Disease Risk/Vaccine Safety**		
Specific illnesses are attributed to vaccination, (eg, multiple sclerosis, autism, asthma, sudden infant death syndrome)	91	0.529
Vaccines contain contaminants, mercury, or there are “hot lots” of vaccine that cause adverse events	83	0.687
Vaccines afford only temporary protection and/or outbreaks occur despite vaccination	79	0.688
Diseases prevented by vaccines have declined, are not contagious, or are relatively mild illnesses	74	0.702
Physicians under-report adverse reactions	62	0.677
**Alternative Medicine**		
Encourages “back to nature” alternatives to vaccination such as homeopathy, vitamins/minerals/supplements, chiropractic services	67	0.565
Conventional medicine is wrong; some physicians disagree with vaccination	63	0.531
Physicians are misinformed about vaccines	58	0.606
Sells herbal and/or homeopathic products	16	0.575
**RHETORICAL APPEAL**		
**Emotive Appeals**		
Responsible parenting mandates educating oneself; parents must stand together against the establishment	76	0.540
Pictures and/or stories of children allegedly harmed by vaccinations	37	0.573
Pictures or diagrams of needles	22	0.522
**Ethical Allegations**		
Safety and efficacy information is false; cover-up and conspiracy about safety is alleged	76	0.528
Civil liberties are violated by taking away parental choice	70	0.666
Conflict of interest exists between vaccine manufacturers and doctors or policy makers	66	0.630
Vaccine mandates infringe on parental rights; totalitarianism is suggested	63	0.561
Immorality argument—vaccines are grown on cell lines derived from abortions; universal vaccination is a form of utilitarianism which sacrifices a few for the benefit of many	46	0.555
Government protects doctors and manufacturers from liability for harm caused by vaccines	33	0.601

*P* < .001 for kappa for each attribute

Of the 25 website characteristics in Table 2, the average number of characteristics per website was 13.5 ± 5.3 (range 1.5–23.5). In order to assess the way in which groups of characteristics were related in vaccine-critical websites, correlation analyses for nonparametric data were performed. Although all were significantly correlated (*P* < .019), the highest correlation coefficients were for the relationships between the ethics group and the disease risk/vaccine safety group (ρ = .637; *P* < .001), the ethics group and the emotion group (ρ = .542; *P* < .001), and the alternative medicine group and the disease risk/vaccine safety group (ρ = .554; *P* < .001). Three content design attributes were identified: 62% of sites contained references to scientific literature (κ = .60; *P* < .001); 28% provided links to vaccine proponents' websites (κ = .68; *P* < .001); 26% provided information on or links to states' immunization requirements (κ = .66; *P* < .001).

## Discussion

We found that websites critical of vaccines claim that vaccines cause illness, claim that vaccines are contaminated, promote the idea that the vaccines are only temporarily effective, encourage alternative medicine, claim conventional medicine is wrong, make emotive appeals, and make ethical allegations about conspiracy, cover-up, civil liberty violations, totalitarianism, and immorality.

The Institute of Medicine reviewed the scientific evidence for a number of vaccine controversies, published multiple texts on the issues, and has generally found vaccines to be safe, albeit with rare risks such as anaphylaxis [18-23]. A published review of the veracity of claims by websites critical of vaccination reports many “fabrications and distortions” and misrepresentation of the data from reputable medical journals [24].

The number of vaccine-critical websites may be increasing. We found 78 sites in 2004, whereas Nasir found 51 sites in 1999, and Wolfe et al found 22 in 2000 [13,14].

### Relativism, Logic Fallacies, and Heuristics

We believe that there is a link between the claims we evaluated about conventional medicine being wrong, about physicians being misinformed about vaccination, and about the promotion of “back to nature” alternatives and homeopathy. These are all common in post-modern thought, which considers truth to be relative and which questions established points of view. Thus, the viewpoint of a homeopath or herbalist may be considered as legitimate, or more legitimate, than the opinion of traditional authorities such as physicians and scientists. Evidence of this was seen in an analysis of parents of unvaccinated children in the National Immunization Survey, which found that 71% said that a doctor is not influential in shaping vaccination decisions for their children [1].

We found that personal stories or pictures of children allegedly injured by vaccines appeared on 37% of websites. Information from the disciplines of logic and debate may help in analyzing and responding to such allegations. The linking of such alleged adverse reactions with vaccination appears to commit two logic fallacies. One is *post hoc ergo propter hoc*, which translates into “occurring afterwards, therefore occurring because,” in other words, confusing temporal association with causality. The second logic fallacy is *faulty dilemma*. In this case, the argument forces a choice between two options, both of which are contrary to a third position, which is not mentioned as an option. For example, given a description of a disabled child, the choice is either the vaccine caused the disability or the child is not disabled; the third option that the disability was genetically determined or occurred in utero is not mentioned as a possibility.

Several other heuristic processes may be involved in parental analyses of vaccine risks, including compression, omission bias, and ambiguity aversion. Compression is the overestimation of rare risks, such as vaccine reactions, but an underestimation of common risks, such as the morbidity and mortality of vaccine-preventable diseases [25]. The news media tend to overemphasize risk of death from infrequent causes and to under-represent risk of death from more common causes [26]. Omission bias is the tendency to favor errors of omission over errors of commission, even though a distinction between them may be irrelevant [27,28]. Ambiguity aversion applies to cases in which parents tend to avoid ambiguity and may find a greater risk from a known disease more acceptable than a smaller, more ambiguous risk from a new vaccine [25,28]. Ambiguity aversion also applies to a situation in which there is debate about the reliability of vaccine information. One study found that those opposed to vaccination were more strongly opposed after being shown a table comparing the risks of pertussis disease with the risks of whole cell DTP (diphtheria, tetanus, pertussis) vaccine, suggesting that they focused on information that supported their previous beliefs even when presented a balanced picture [28].

### Ethical Allegations

The ethical allegations of conspiracy, cover-up, civil liberty violations, totalitarianism, and immorality that we found frequently in websites critical of vaccination are particularly troubling, given the serious nature of the charges. The handling of the rare cases of intussusception following vaccination with rhesus monkey-derived rotavirus vaccine (RRV) challenges the conspiracy and cover-up allegations. In this case, personnel from the Centers for Disease Control noted a signal in the vaccine adverse events reporting system (VAERS), instituted a study, and rapidly found an association between RRV and intussusception. RRV was withdrawn within weeks [29-31].

Exemptions to states' mandatory vaccination laws are a counter-argument to the aforementioned ethical allegations. Although state laws require vaccination prior to school entry, all states allow exemptions for medical reasons, 48 allow them for religious reasons, and 17 for philosophical reasons [32]. States that allow philosophical exemptions to laws mandating vaccination for school entry have significantly higher rates of unvaccinated children [1].

An analysis of vaccine immorality allegations based on the fact that a few vaccines are grown in self-propagating cell lines originally obtained from two abortions in the 1960s was recently published [33]. The paper used strategies to analyze moral complicity (eg, principle of double effect) and found that vaccination is ethical [33]. The abortions were past events separated in time, agency, and purpose from vaccine production. Indeed, the ethics of altruism and herd immunity argue for widespread vaccination, although concerns about autonomous decisions and personal conscience should be respected [33].

### Historical Context

Since the introduction of smallpox vaccine and compulsory vaccination, there have been small but vocal movements against vaccinations which share many similarities with criticisms of the past. First, vaccine criticism of the past and present capitalizes on the public's lack of understanding of medical science and investigation and their limited ability to confirm or refute claims. The general public is not skilled in interpreting statistical results, in differentiating between causality and temporal association, or in assessing the validity of findings based on appropriate study design. Second, many of the arguments in use today parallel those used in the past. For instance, during the late 19th century, objections to smallpox and typhoid vaccinations included the following: vaccination is against the laws of nature, good hygiene provides adequate protection against disease, vaccines can transmit other diseases, and compulsory vaccination is a violation of one's liberty [34,35]. These arguments are similar to those espoused by current vaccine critics who hold that natural therapies and alternative medicine are preferable for prevention of infectious disease, vaccines cause idiopathic illness, and school entry vaccination requirements violate civil liberties [13,14]. Furthermore, the ethical allegations remain quite strident, including purported collusion among government, the medical establishment, and pharmaceutical companies that is motivated by profit [35]. Finally, opponents of vaccination dramatize relatively rare adverse events to overshadow vaccination's enormous public health benefits [15]. This is an especially effective tactic now, as the toll from a number of infectious diseases fades from the public memory (as a result of universal vaccinations).

Differences between vaccine criticism of today and the past are principally a matter of degree. There are now more vaccines and therefore more available to criticize. Secondly, there are many more resources for dissemination of health information, including television, radio, and the World Wide Web.

### Strengths and Limitations

This is the largest study conducted by US investigators on this topic and the most complete and current in the literature. In addition, our design builds on prior studies by quantifying ethical allegations on the reviewed websites.

As was the case in prior studies, non-English sites were not reviewed, which limits the ability to generalize results. Also, interrater reliability was good but not excellent. We believe that this primarily reflects inherent individual differences in the interpretation of website content when determining the presence or absence of value-related issues such as conspiracy, immorality, and civil liberties violations. The complexity and size of websites are other factors that may have affected the interrater reliability.

### Solutions

There are several strategies to encourage openness to vaccination among parents who are concerned about the risk of causing their children harm from vaccines. These strategies can be used in mass education campaigns or in discussions between a clinician and parents. One strategy is to share personal experiences with diseases such as pertussis, which can cause serious illness and disability and which still circulates in the United States. Pictures [36,37] and testimonials [38] of children suffering from vaccine-preventable diseases may be helpful.

A second strategy is to explain the communicable nature of most vaccine-preventable diseases and their recurrence in industrialized countries when vaccination rates decline. For instance, pertussis returned after immunization rates decreased in Sweden, England, Wales, and Japan [39-41]. Third, some websites that are critical of vaccination sell products, including homeopathic and herbal products, raising the possibility of conflict of interest in these particular sites—an important point to raise with parents. Finally, non-profit websites such as the Vaccine Education Center [42] and the National Network for Immunization Information [43] provide useful information for parents and providers that is free from commercial and federal funding.

### Conclusions

In summary, websites critical of vaccines allege serious adverse reactions, vaccine failure, and serious ethical violations, including cover-up, conspiracy, and civil liberties violations. As physicians encounter an increasing number of parents and patients who have searched the Internet for vaccine information, they need to be aware of the medical and ethical allegations being made against vaccination. Strategies such as encouraging parents to take the child's perspective, sharing the physician's experience of treating patients with vaccine-preventable diseases, and providing pictures and testimonials of persons affected by vaccine-preventable diseases may be useful.
